# EARLY INCISIONAL HERNIA AFTER LIVER TRANSPLANTATION: RISK FACTORS AND HERNIA REPAIR RESULTS

**DOI:** 10.1590/0102-672020220002e1698

**Published:** 2022-11-04

**Authors:** João Victor Vecchi Ferri, Sofia Michele Dick, Tomaz de Jesus Maria Grezzana-Filho, Flávia Heinz Feier, Lucas Prediger, Glória Sulczinski Lazzaretti, Cleber Rosito Pinto Kruel, Carlos Otavio Corso, Leandro Totti Cavazzola, Marcio Fernandes Chedid

**Affiliations:** 1 Militar Hospital of Porto Alegre, Surgery Unit – Porto Alegre (RS), Brazil; 2 University Hospital of Porto Alegre, Surgery Unit – Porto Alegre (RS), Brazil; 3 Federal University of Rio Grande do Sul, Faculty of Medicine – Porto Alegre (RS), Brazil.

**Keywords:** Liver Transplantation, Incisional Hernia, Abdominal Wall, Risk Factors, Postoperative Complications, Transplante de Fígado, Hérnia Incisional, Parede Abdominal, Fatores de Risco, Complicações Pós-Operatórias

## Abstract

**BACKGROUND:**

Liver transplantation is a complex and valuable therapy. However, complications that burden postoperative quality of life, such as incisional hernia, are to be better elucidated, such as risk factors and prophylactic measures.

**AIM:**

This study aimed to define the rate of incisional hernia in patients who underwent liver transplantation in a population in southern Brazil and to assess the related risk factors in order to establish measures for prior optimization and specific prophylactic care in the future.

**METHODS:**

Patients undergoing adult Liver transplantation from January 2004 to November 2020 were retrospectively analyzed, assessing demographic features, surgical outcomes, and predisposing factors.

**RESULTS:**

Among 261 liver transplantation patients included, incisional hernia was diagnosed in 71 (27.2%). Of the 71 incisional hernia patients, 28 (39.4%) developed IH during the first post-transplant. Majority of the patients were male (52/71, 73.2%); of the 71 patients, 52 had hepatitis C virus (HCV) and 33 (46.5%) had hepatocellular carcinoma (HCC). Male gender (p=0.044), diabetes mellitus (p=0.008), and acute cellular rejection (p<0.001) were risk factors for IH. In all, 28 (39.4%) patients were submitted for hernia repair with mesh, with a recurrence rate of 17.8%.

**CONCLUSION:**

Incisional hernia after liver transplantation is a relatively common problem associated with male gender, diabetes, and acute cellular rejection. This is a problem that should not be trivialized in view of the complexity of liver transplantation, as it can lead to a reduction in quality of life as well as jeopardize late liver transplantation results and lead to incarceration and strangulation.

## RESUMO

**RACIONAL:**

O transplante de fígado é uma terapia complexa e valiosa. Entretanto, complicações que prejudicam a qualidade de vida pós-operatória, como a hérnia incisional, devem ser mais bem elucidadas, analisando os fatores de risco e medidas profiláticas.

**OBJETIVOS:**

Definir a taxa de hérnia incisional em pacientes submetidos a transplante de fígado em uma população do sul do Brasil, avaliar os fatores de risco relacionados, a fim de estabelecer futuramente medidas de otimização prévia e cuidados profiláticos específicos.

**MÉTODOS:**

Foram analisados, retrospectivamente, pacientes submetidos a transplante de fígado adultos, de janeiro de 2004 a novembro de 2020, avaliando suas características demográficas, resultados cirúrgicos e fatores predisponentes.

**RESULTADOS:**

Dentre os 261 pacientes transplantados hepáticos incluídos, a hérnia incisional foi diagnosticada em 71 (27,2%). Vinte e oito do total de 71 pacientes com hérnia incisional (39,4%) desenvolveram hérnia incisional durante o primeiro ano pós-transplante. A maioria era do sexo masculino [n=52, (73,2%)]; 52/71 (73,2%) apresentavam cirrose secundária ao vírus da hepatite C; 33/72 (46,5%) foram portadores de carcinoma hepatocelular. Sexo masculino (p=0,044), diabetes mellitus (p=0,008) e rejeição celular aguda (p<0,001) foram fatores de risco estatisticamente significantes para hérnia incisional. Vinte e oito pacientes (39,4%) foram submetidos à hernioplastia incisional com tela, com taxa de recidiva de 17,8%.

**CONCLUSÕES:**

Hérnia incisional após transplante de fígado é um problema relativamente comum, associado ao sexo masculino, diabetes e também a rejeição celular aguda. Este é um problema que não deve ser banalizado, já que pode levar à redução da qualidade de vida, comprometer os resultados tardios do transplante de fígado e pode levar a encarceramento ou estrangulamento.

## INTRODUCTION

Liver transplantation (LT) is acknowledged to be the only definitive treatment option for patients with end-stage liver disease^6^, and some cases of unresectable hepatic neoplasms^2^. With advances in the results of this therapy, greater safety, and prolonged patient survival, the focus is increasingly on the quality of life of the recipients.

As these individuals sometimes have numerous preconditions for general postoperative complications, such as diabetes mellitus, advanced age, obesity, and malnutrition^10,22^, in addition to the specific debilitating characteristics of patients with liver disease, these outcomes may impair LT results.

Among these outcomes are incisional hernias (IH), with an incidence of around 4–20%^23,25^, in some cases disabling and reducing the quality of life of patients, and also related to a greater rate of hospitalization and emergency surgeries, especially in cases of incarceration and strangulation, putting at risk a therapy as complex and valuable as LT.

The main objective of this study was to define the rate of IH in patients who underwent LT in a population in southern Brazil and to assess the related risk factors in order to establish measures for prior optimization and specific prophylactic care in the future. Secondarily, cases that underwent IH repair with mesh are described, as well as cases of hernia recurrence after the correction procedure.

## METHODS

All patients undergoing adult LT from January 2004 to November 2020 were retrospectively analyzed. The observation period was 12 months. Patients with a second transplant or more, or who did not have the abdominal wall completely closed in LT for a variety of reasons (e.g., critical patients) or who died within the first year or lost outpatient follow-up of at least 1 year, were excluded, and the remaining 261 patients were included in the study.

The minimum age was 18 years. The main incision used was the bilateral subcostal transverse with superior median extension (“*Mercedes incision*”). The musculature was closed in at least two muscle layers with either polyglycolic acid, polyglycolic acid *plus* polypropylene, polypropylene, or polydioxanone, with size 0 or 1. The skin was closed most commonly with nylon 3–0 and the stitches were removed on postoperative day 30.

Antibiotic prophylaxis was cefuroxime plus vancomycin for 72 h. The initial immunosuppressive therapy consisted mainly of tacrolimus, mycophenolate, and corticosteroid, and lasted for only 6 months.

Demographic characteristics, including hypertension, diabetes mellitus, clinical ascites, underlying diseases such as hepatitis C virus (HCV) and hepatocellular carcinoma (HCC), albumin and hematocrit before LT, Child-Pugh and ALBI (albumin-bilirubin) score, body mass index (BMI), MELD-Na (Model for End-stage Liver Disease-Na) score, blood loss during LT, postoperative infection, abdominal reoperation within 3 months of LT, and type of suture for musculoaponeurotic closure, were collected. Acute cellular rejection (ACR) was analyzed mainly by liver biopsy results. Hernia was diagnosed either during a physical examination of outpatient control or by radiology (e.g., computed tomography and control ultrasound). The outcomes of IH, hernia repair, and hernia recurrence were further evaluated.

Statistical analyses were performed using the SPSS (PASW Statistics for Windows, version 18.0; SPSS Inc., Chicago, IL, USA) software. Categorical variables, presented as N (%), were compared using the chi-square test and the Mann-Whitney U test. Continuous variables were analyzed using the Mann-Whitney U test, presented as median (interquartile range), and Student’s t-test, presented as mean (standard deviation). Differences with a p-value <0.05 were considered statistically significant for all comparisons. The study was approved by the Ethics Committee under the number 2017-0271, by the University Hospital of Porto Alegre, Rio Grande do Sul (RS), Brazil.

## RESULTS

From 381 patients submitted to LT during the study period, 120 were excluded based on exclusion criteria [including 16 (4.1%) individuals due to a second LT and a previous main incision], leaving 261 patients for analysis. Age ranged from 18 to 71 years, with a median of 57.5 (14.5) years. The main causes of LT were cirrhosis due to HCV and HCC.

Notably, 71 (27.2%) patients developed IH during the follow-up year, while the other 190 (72.7%) had no IH, composing two study groups. Of the total 71 IH patients, 28 (39.4%) developed IH during the first post-transplant year: 7 (9.8%) in the first 90 post-transplant days, 9 (12.6%) patients developed IH from 90^th^ to 180^th^ post-transplant days, and 12 (16.9%) patients had IH from 180^th^ to 365^th^ post-transplant days. Of the 71 patients with IH, 14 (19.7%) were diagnosed during the second post-transplant year, 12 (16.9%) had IH revealed on the third post-transplant year, 7 (9.8%) had IH revealed on the fourth post-transplant year, and 2 (2.8%), 3 (4.2%), and 2 (2.8%) patients had the diagnosis of IH, respectively, on the fifth, sixth and seventh post-transplant year. The remaining 3 (4.2%) patients had the diagnosis of IH after completing 7 years of post-transplant follow-up.

The two study groups had no significant differences concerning age, hypertension, clinical ascites, immunosuppressive agents (such as mTOR inhibitors), ALBI scores, liver diseases (i.e., HCV and HCC), MELD-Na score, pretransplant albumin and hematocrit, obesity, blood loss during LT, postoperative infection, abdominal reoperation in 3 months after LT, or suture material for closure (Table 1).

**Table 1:** Demographic and surgical characteristics of the study population.

Parameter		No incisional hernia	Incisional hernia	p-value
n (%)		190 (72.7%)	71 (27.3%)	
Male (%)		112 (68.3%)	52 (31.7%)	0.044
Age, median (IQR)		57.4 (15.3)	57.4 (12.1)	0.996
Hypertension (%)		50 (69.4%)	22 (30.5%)	0.454
Diabetes (%)		46 (61.3%)	29 (40.8%)	0.008
Clinical ascites (%)		75 (71.4%)	30 (28.5%)	0.685
HCV (%)		118 (69.4%)	52 (30.6%)	0.143
HCC (%)		101 (75.3%)	33 (24.6%)	0.488
MELD-Na, median (IQR)		15.0 (10)	15.0 (11)	0.585
Child-Pugh score				0.129
	A	73 (80.2%)	18 (19.7%)	
	B	64 (67.3%)	31 (32.6%)	
	C	53 (70.6%)	22 (29.3%)	
ALBI score				0.522
	Grade 1	22 (81.4%)	5 (18.5%)	
	Grade 2	113 (72.9%)	42 (27.1%)	
	Grade 3	54 (70.1%)	23 (28.5%)	
Hematocrit, mean (SD)		33.87 (6.56)	32.93 (6.57)	0.303
Albumin, mean (SD)		3.26 (0.65)	3.17 (0.55)	0.251
Obesity		39 (68.4%)	18 (31.6%)	0.396
Blood loss, median (IQR)		2,406.5 (2,450)	2,667.0 (2,109)	0.189
Postoperative infection		73 (70.9%)	30 (29.1%)	0.670
Abdominal reoperation in 3 months		30 (62.5%)	18 (37.5%)	0.105
mTOR inhibitor (%)		16 (88.8%)	2 (11.1%)	0.117
Acute cellular rejection (%)		13 (31.7%)	28 (68.3%)	<0.001
Suture material				0.821
Polyglycolic acid		130 (73.4%)	47 (26.6%)	
Polyglycolic acid *plus* polypropylene		17 (73.9%)	6 (26.1%)	
Polypropylene		17 (65.4%)	9 (34.6%)	
Polydioxanone		25 (75.8%)	8 (24.2%)	

HCC: hepatocellular carcinoma; HCV: hepatitis C virus; MELD: model for end-stage liver disease; ALBI: albumin; SD: standard deviation; IQR: interquartile range; Na: sodium; mTOR: immunosuppressive agent.

In all, 52 (31.5%) of 165 male patients and 19 (19.8%) of 96 female patients developed IH. A significant association was confirmed for male gender (p=0.044).

Diabetes mellitus was present in 75 (28.7%) patients in the study and was also significantly associated with the incidence of IH (p=0.008).

Comparing the IH group versus the control group, the albumin level was lower (3.17 vs. 3.26), hematocrit was lower (32.93 vs. 33.87), and they bled more during the LT (2.667 vs. 2.406 mL), but not significant statistically. There were no significant differences in relation to the suture material among the groups.

ACR of the liver developed in 41 (15.7%) patients is associated with IH (p<0.001).

Of the 71 patients who developed IH, 28 (39.4%) were submitted for hernia repair. In all cases, a prosthetic mesh was placed, most commonly in an onlay position (71.4%). Five (17.8%) patients developed hernia recurrence at some point in their follow-up. All of them had HCV as an etiology of LT, and 4 (80%) were male (Table 2).

**Table 2:** Hernia repair results.

Technique	Hernia repair	Recurrence by technique
n	28	5
Onlay	20	3
Sublay	4	1
Inlay	1	1
Intraperitoneal	1	0

## DISCUSSION

IH is a common complication after LT^19^. The results showed an incidence of 27.2%, above the average of other studies^3^, around 15.1%.

This is a complication that should be valued, as hospitalizations independently lead to reduced graft and patient survival^3^. In this way, it is increasingly searched to identify the factors associated with postoperative complications in patients undergoing LT.

There are well-established risk factors in the general surgical population, such as age over 45 years, obesity, surgical wound infection, previous abdominal surgery, diabetes mellitus, chronic lung disease, and nutritional deficiencies^25^. As these characteristics are often present in transplant patients, adding to the entire burden of ongoing liver disease, these individuals are the strong candidates for IH.

For example, the use of steroids and immunosuppressive agents, ascites, and even lack of surgeon experience has been described as risk factors^9^. In addition, end-stage liver disease is usually complicated with cachexia and muscle wasting^17,18^. Patients with chronic liver disease often exhibit protein-energy malnutrition due to hypermetabolism and malabsorption. Furthermore, ascites, often present as a consequence of portal hypertension, increases intra-abdominal pressure and contributes to abdominal muscle weakness^5,12^.

There are other risk factors found in the literature, such as the use of sirolimus and mycophenolate for immunosuppression, end-stage liver disease (Child C), male gender, surgical wound infection, severe ascites, BMI >25, age over 55 years, low platelet count, and cirrhosis caused by viruses^4,11,12,16,19,21^.

The use of steroids and immunosuppressants is already well established after LT for immune response control. However, steroids negatively influence healing, and the use of mycophenolate and sirolimus is also related to poorer healing quality, unlike tacrolimus^1,14^. Thus, these may be some of the most potent and modifiable factors for preventing IH^3^. ACR was associated with IH in the present study, perhaps mainly due to the increased use of steroids for its treatment. However, Cos et al.^4^ found ACR to have an inverse association with IH, mainly due to the better immune system and nutritional status of these patients, leading to the immune response.

Regarding the type of incision used, Gastaca et al.^8^ that the right subcostal incision with an extension to the xiphoid process (J-shaped) was related to a lower rate of IH than the “*Mercedes incision.*” In the latter, the incision area is larger and there is a central area susceptible to a greater risk of ischemia and consequently worse healing.

Hernia patients can suffer chronically and/or acutely. In the first, reducing the quality of life in the long term, whether due to chronic pain or making it difficult to practice physical activities, such as weight training, with consequent worse maintenance of health and functionality (sedentary lifestyle and cardiovascular disease). In the second, through emergency care due to incarcerated and sometimes strangulated hernias, putting at risk the results of the LT and the patient’s own life, as the risk of emergency surgeries is greater in this population. Furthermore, intestinal obstruction can rapidly affect the levels of immunosuppressive agents, leading to toxicity and renal failure^3^.

Treatment involves surgical correction of hernias with the use of meshes to reinforce the musculoaponeurotic system and reduce recurrence rates. Thus, the aim is to reestablish the functionality of the abdominal wall of these patients and increase their quality of life^24^.

It is important to remember that these procedures, in this population, can occur in a context of previous/chronic active infections, loss of domain, intestinal involvement, and other significant comorbidities^20^, such as immunosuppression; in addition, some of these are hernias with wide bidirectional incisions (e.g., “*Mercedes incision*”), hence the importance of better studying this population subgroup within the hernia universe. However, mesh repair is still an efficient and safe method for LT patients, not associated with increased morbidity, despite continuous use of immunosuppressants^7,15^.

Regarding the rise of IH, we reported a rate of 17.8%, above the rate reported in the literature of 12.4%^3^. The size of the hernia and the use of steroids are related to a higher risk of hernia recurrence^13^, characteristics often present in LT recipients. The use of meshes is recommended, as they reduce the risk of recurrence and are not necessarily related to increased rates of infection and other operative wound problems^25^.

## CONCLUSION

IH after LT is a relatively common problem, which is more associated with males and patients with diabetes, and to ACR. In addition, these patients have several general risk factors for the rise of IH, such as malnutrition, as well as specific factors, such as the use of steroids, immunosuppressants, and ascites. Treatment involves surgical correction of hernias with the use of meshes to reinforce the musculoaponeurotic system and reduce recurrence rates. This is a problem that should not be trivialized in view of the complexity of the previous LT, as it can reduce quality of life as well as jeopardize LT results and risks of emergency surgeries.
